# Estimated cost of treating hospitalized COVID-19 patients in Saudi Arabia

**DOI:** 10.1038/s41598-022-26042-z

**Published:** 2022-12-12

**Authors:** Abbas Al Mutair, Laila Layqah, Batool Alhassan, Saleh Alkhalifah, Modhahir Almossabeh, Thanaa AlSaleh, Zuhair AlSulaiman, Zainab Alatiyyah, Eman M. Almusalami, Lamiaa H. Al-Jamea, Alexander Woodman, Khalid Hajissa, Saad Alhumaid, Ali A. Rabaan

**Affiliations:** 1 Research Center, Almoosa Specialist Hospital, Al-Ahsa, Saudi Arabia; 2 School of Nursing, University of Wollongong, Wollongong, Australia; 3 Princess Norah Bint Abdulrahman University, Riyadh, Saudi Arabia; 4 Nursing Department, Prince Sultan Military College, Dhahran, Saudi Arabia; 5 Nursing Department, Almoosa College of Health Sciences, Al-Ahsa, Saudi Arabia; 6 Research Office, King Abdullah International Medical Research Center, Riyadh, Saudi Arabia; 7 King’s College London, Strand, London, WC2R 2LS UK; 8 Vice Deanship of Postgraduate Studies and Research, Prince Sultan Military College of Health Sciences, Dhahran, Saudi Arabia; 9 Department of Medical Microbiology and Parasitology, School of Medical Sciences, University Sains Malaysia, 16150 Kubang Jerian, Kelantan Malaysia; 10 Administration of Pharmaceutical Care, Al-Ahsa Health Cluster, Ministry of Health, Al-Ahsa, Saudi Arabia; 11 Molecular Diagnostics Laboratory, Johns Hopkins Aramco Healthcare, Dhahran, Saudi Arabia; 12 College of Medicine, Alfaisal University, Riyadh, Saudi Arabia; 13 Department of Public Health and Nutrition, The University of Haripur, Haripur, Pakistan

**Keywords:** Diseases, Health care, Medical research

## Abstract

The economic impact of the COVID-19 pandemic on global health systems is a major concern. To plan and allocate resources to treat COVID-19 patients and provide insights into the financial sustainability of healthcare systems in fighting the future pandemic, measuring the costs to treat COVID-19 patients is deemed necessary. As such, we conducted a retrospective, real-world observational study to measure the direct medical cost of treating COVID-19 patients at a tertiary care hospital in Saudi Arabia. The analysis was conducted using primary data and a mixed methodology of micro and macro-costing. Between July 2020 and July 2021, 287 patients with confirmed COVID-19 were admitted and their data were analyzed. COVID-19 infection was confirmed by RT-PCR or serologic tests in all the included patients. There were 60 cases of mild to moderate disease, 148 cases of severe disease, and 79 critically ill patients. The cost per case for mild to moderate disease, severe disease, and critically ill was 2003 USD, 14,545 USD, and 20,188 USD, respectively. There was a statistically significant difference in the cost between patients with comorbidities and patients without comorbidities (*P-*value 0.008). Across patients with and without comorbidities, there was a significant difference in the cost of the bed, laboratory work, treatment medications, and non-pharmaceutical equipment. The cost of treating COVID-19 patients is considered a burden for many countries. More studies from different private and governmental hospitals are needed to compare different study findings for better preparation for the current COVID-19 as well as future pandemics.

## Introduction

COVID-19 is an ongoing global pandemic that caused a huge disturbance in healthcare systems in most countries^1^. In addition, it severely affected the global economy^2^. The number of COVID-19-infected patients increased sharply at the beginning of the pandemic in which hospitals and healthcare systems faced great challenges to control the situation. The economic impact of the COVID-19 pandemic on global health systems is a major concern; there was an urgent need for additional resources and financial investments^3^. Financial challenges related to the pandemic COVID-19 affect most hospitals and healthcare facilities^1^.

Even though recovery signs from the COVID-19 pandemic starts to appear, efforts are needed for a full restoration of the previous normal life^1^. COVID-19 is not likely to disappear shortly^2^. Therefore, to manage the progression of the pandemic COVID-19 appropriately, healthcare systems should be aware of the required resources and measures. These resources are needed for education, screening, testing, isolation, and treating patients in general wards as well as in intensive care units (ICU)^4^.

Based on WHO, COVID-19 claimed more than six million lives^5^. According to the National Audit Office, public spending on COVID-19-related measures was estimated to be £260 bn^6^. Healthcare support was budgeted at £84.3bn; accounting for 23% of the total which was the second largest area of spending after the spending for business support^6^. Around £17.9bn was dedicated to the tests and trace program, £13.8bn for the procurement of personal protective equipment, £7.8bn was spent by the NHS, and £1.8bn for vaccine and antibody supply^7^. Furthermore, according to the International Monetary Fund World Economic Outlook Update, the estimated cumulative output loss since the start of the pandemic through 2024 is estimated to be $13.8 trillion^8^. In addition, the financial burden of the pandemic COVID-19 has many other different reasons. Europe reported that there was an average of 7.4% reduction in the gross domestic product in 2020 varying between different European countries^9^. Lockdown affects tourism especially for countries depending on tourism for their economy. Moreover, a reduction in the employment rate resulted in some people do not have enough money to eat, pay the rent, and live as pre-COVID-19 life. Additionally, people’s death including healthcare providers is associated with direct, indirect, and tangible cost.

Mitigation measures affect the Saudi economy by decreasing oil demand and airline services, decreasing manufacturing functions and supply chains, and disrupting religious tourism^10^. The Health system in Saudi Arabia adopted many strategies to combat the pandemic COVID-19 with the least possible economic damage. At the beginning of COVID-19, the Saudi government allocated an emergency budget of US$ 32 billion^10^. Early intervention and application of national mitigation measures across the kingdom before the first COVID-19 case detection was the strongest strategy to avoid future collapse^10^. After that, Saudi MOH started quarantining of epidemic areas, travel restrictions, expansion of serological screening, mask-wearing, and social distancing along with disseminating information regarding the virus for awareness and educational purposes^10^. To advocate for human values, Saudi Arabia’s Ministry of Health decided to provide medical treatment for all citizens and residents infected with COVID-19 free without any charge^10^.

Pandemics and epidemics will continue to occur leading to global challenges to lives, societies, and countries’ economies^11^. The resources used to support an emergency crisis such as Ebola, SARS, and COVID-19 have a financial burden on the country’s government; affecting the way a country conducts its budget. Financial consideration is needed to be studied by the government for policy making to have a clear plan for emergencies. This is to release the pressure on the government economy and reduce economic uncertainty. As a lesson learned from the pandemic COVID-19, pandemic preparedness through engaging the stakeholders and policymakers is deemed necessary to reduce unnecessary struggles associated with pandemics^12^.

Understanding COVID-19-specific medical costs are critical, especially for healthcare providers, insurance payers, and the Saudi healthcare system to provide the required information to plan and allocate resources to treat COVID-19 patients^12^. Moreover, it gives insights into the financial sustainability of the Saudi healthcare system in fighting the future pandemic^13^. There is only one study in Saudi Arabia that measured the average direct medical cost for COVID-19 patients. Therefore, we conducted this study to measure the direct cost required to treat COVID-19 patients with different disease severities and different clinical statuses.

## Method

### Study design

It is a retrospective, real-world observational study to measure the direct cost of treating COVID-19 patients. The evaluation was conducted for all symptomatic patients with confirmed COVID-19 after being tested in inpatient setting. Data were collected from a private hospital in Saudi Arabia between July 2020 and July 2021. The Hospital is a 500-bed tertiary care center, serving a local catchment population of over 1.4 million people with all medical specialties available. Exclusion criteria were COVID-19 vaccinated patients, and patients treated in an outpatient setting.

To calculate the minimum required sample size, Walters’s formula for non-normally distributed continuous data were applied. In this calculation, a two-tailed 5% significance level, effect size (P_Noether_) of 0.51 (consistent with those used in common association analyses), 80% power, and response rate of 80% was considered, which gives the estimated number of subjects as 287.

$$n = \frac{{2\left( {Z_{{1 - {\raise0.7ex\hbox{$\alpha $} \!\mathord{\left/ {\vphantom {\alpha 2}}\right.\kern-\nulldelimiterspace} \!\lower0.7ex\hbox{$2$}}}} + Z_{1 - \beta } } \right)^{2} }}{{6\left( {P_{Noether} - 0.5} \right)^{2} }}$$

### COVID-19 severity classification

According to the Saudi Ministry of Health Protocol for Patients Suspected of/Confirmed with COVID-19, (Version 3.6), April 14th, 2022^7^, disease severity can be classified as follow:

1. Mild disease
  Symptomatic patients meet the case definition for COVID-19 without evidence of viral pneumonia or hypoxia.
2. Moderate disease/Pneumonia
  Adult with clinical signs of pneumonia (fever, cough, dyspnea, fast breathing).
3. Severe disease/Severe pneumonia
  Adult with clinical signs of pneumonia (fever, cough, dyspnea, fast breathing) plus one of the following conditions: (i) respiratory rate > 30 breaths/min. (ii) severe respiratory distress; or oxygen saturation ≤ 93% on room air. iii) ratio of partial pressure arterial oxygen and the fraction of inspired oxygen ≤ 300 mm Hg.
4. Critically ill

Presence of any of the following conditions: (i) respiratory failure requiring mechanical ventilation. (ii) Shock. (iii) another organ failure that requires monitoring and treatment in an intensive care unit (ICU).

### Data collection

The analysis was conducted using primary data and a mixed methodology of micro and macro-costing. The resources used by each patient were identified and quantified using electronic prescriptions, and valued using hospital supply unit information to allow for the determination and description of individual admission costs. Drugs, laboratory testing, radiologic exams, blood components, and feeding requirements were all direct-cost subcategories of micro-costing for individual admission expenses. The direct costs of hospital supply inwards, emergency departments and ICUs, including general supplies and personal protective equipment were considered macro-costing.

All patients were treated according to the updated Clinical Management Guideline for COVID-19 that was developed and regularly updated by the Saudi Ministry of Health.

The variables extracted were age, sex, comorbidities, medications used, laboratory and imaging tests, medical procedures, date of hospital admission, date of discharge from hospital, inpatient environment (ICU vs. General Medical Ward (GMW)), and clinical outcome (death vs. discharge).

Input data and their associated quantities for each treatment pathway were estimated based on the classification criteria guidelines. The costs were recorded in Saudi Arabia Riyals (SAR) and converted into US dollars (USD) using an exchange rate (as of 30th March 2021); 1 USD was worth an average of 3.75 SAR.

### Ethical consideration

An ethical clearance to conduct the study was obtained from the Institutional Review Board of Almoosa Specialist Hospital (IRB log Number: ARC-21.11.01). Informed consent was waived by the Almoosa Specialist Hospital IRB as the study was retrospective, and the data were de-identified for the use of this publication. All research procedures were performed in accordance with the Declaration of Helsinki.

### Statistical analysis

Statistical analyses were conducted using the statistical software SPSS 24.0 (IBM Corp, Armonk, NY). The continuous variables were expressed as mean with standard error (SE), and median with interquartile range (IQR) for all sociodemographic and clinical subgroups. Categorical variables were expressed as the number of cases and percentages. Shapiro–Wilk test was used to test the distribution of the data. The non-parametric Mann–Whitney and Kruskal–Wallis tests were used to statistically compare the differences for two, and more than two groupsdeviations, respectively. Descriptive analysis was conducted to compare the cost stratified to different patient groupsthe a. A *P*-value less than 0.05 was considered significant.

## Results

Between July 2020 and July 2021, 287 patients with confirmed COVID-19 were admitted. The average age for the included subject was 59 years, and 55% of the participants were male. COVID-19 was confirmed by reverse transcription–polymerase chain reaction (RT-PCR) or serologic tests in all the included patients. Only 58 (20%) patients of the admitted patients had no comorbidities while the rest of the patients had one or more comorbidities. The most frequent comorbidities were diabetes mellitus (54%), followed by cardiac diseases (53%), then renal disease (20%). Around half of the included patients were classified as having severe COVID-19 and 27.5% of the included patients were admitted to ICU (Table 1).

**Table 1:** Demographic and clinical characteristics of patients.

Characteristic	Patients (n = 287) n (%)
Age, Mean ± SD	59.2 ± 15.9
Gender	
Male	158 (55)
Female	129 (45)
Pre-existing disease*	
None	58 (20.2)
Neurological Disease	18 (6.3)
Cardiovascular disease	153 (53.3)
GI disease	7 (2.4)
Renal disease	56 (19.5)
Respiratory disease	30 (10.5)
Malignancy disease	6 (2.1)
Endocrine disease	27 (9.4)
Diabetes mellitus	155 (54)
Hematological disease	14 (4.9)
*A patient could have more than one comorbidity	
Disease severity	
Mild–Moderate	60 (20.9)
Severe	148 (51.6)
Critically ill	79 (27.5)
Treatment medications*	
Antiviral	207 (72.1)
Antibiotic	266 (92.7)
Antifungal	9 (3.1)
Immunomodulators	80 (27.9)
Anticoagulants	268 (93.4)
Steroid	119 (41.5)
Immunosuppressant	10 (3.5)
Tocilizimub	19 (6.6)
*A patient can receive more than one line of treatment	
Length of Hospital stay	Median, IQR, 8 (9)
	Mean (SE), (12.24 (0.77)
Clinical outcome	
Alive	240 (83.6)
Died	47 (16.4)

The impact of age, gender, comorbidities, and severity of the disease on hospital costs are shown in Table 2. The findings indicated that hospital cost was statistically significant among participants in different age groups (*P* < 0.001). Moreover, the cost per case was significantly higher in critically ill patients compared to patients with mild to moderate COVID-19 (*P* < 0.001). The mean cost for treating mild to moderate, severe, critically ill COVID-19 patients was 2003 USD, 14,545 USD, and 20,188 USD, respectively. The total mean cost for clinical management of COVID-19 according to the presence or absence of other underlying diseases was summarized in Table 3.

**Table 2:** Cost for clinical management of COVID-19 patients stratified by various demographic and clinical characteristics.

Characteristics	Cost per case (USD, Mean ± SE)	Median (IQR)	*P* value
**Sex**			
Male	12,512 (2062)	4438 (8045)	0.17
Female	14,658 (6970)	3806 (6148)	
**Age group (years)**			
18–34	2592 (789)	1770 (3123)	< 0.001
35–60	16,592 (7563)	4092 (7535)	
> 60	12,787 (2221)	4971 (7507)	
**Comorbidities**			
Yes	20,662 (1534)	2760 (4409)	0.006
No	11,656 (1550)	4670 (7858)	
**Severity**			
Mild–Moderate	2003 (266)	1493 (2059)	< 0.001
Severe	14,545 (6230)	4021 (5066)	
Critically ill	20,188 (2859)	10,750 (17,558)

**Table 3:** Comparative cost analysis for clinical management of COVID-19 cases with and without underlying diseases.

Classification	Without underlying diseases		With underlying diseases		*P-*value
	Cost Mean (SD) USD	Median (IQR)	Cost Mean (SD) USD	Median (IQR)	
Accommodation (Bed cost)	315 (70)	134 (229)	892 (197)	200 (354)	0.028
Laboratory works	655 (169)	123 (707)	2517 (642)	312 (1354)	0.031
Diagnostic radiology exams	486 (321)	7.2 (107)	388 (55)	49 (287)	0.159
Treatment Medications	1040 (118)	429 (1143)	17,388 (1679)	208 (867)	The
Nonpharmaceutical (devices, fluid, Intubation, monitoring, and equipment in ICU)	2246 (533)	1218 (2844)	6950 (935)	2611 (5367)	0.002

The mean cost for bed accommodation, laboratory works, treatment medications, and non-pharmaceutical equipment in ICU was significantly higher in patients with comorbidities than in patients without comorbidities (*P* < 0.05). However, the mean cost for the diagnostic radiology exams for both groups was not significantly different (*P* 0.159). The mean cost of a bed was three times higher in patients with underlying diseases compared to patients without underlying diseases; 315 USD, and 892 USD, respectively. Laboratory work’s mean cost was 655 USD for patients without underlying diseases while the mean cost was 2517 USD for patients with underlying diseases. Diagnostic radiology exams’ mean cost between the two groups was less than 100 USD difference. While the mean cost of the treatment medications for patients with underlying comorbidities was 17,388 USD, the mean cost for the medications used for patients without diseases was 1040 USD only. Nonpharmaceutical devices and equipment mean cost was three times more in patients with the underlying disease compared to patients without underlying diseases; 2246 USD, and 6950 USD, respectively. Table 4 presented the estimated financial burden to the national health insurance for COVID-19 patients.

**Table 4:** The estimated financial burden to the national health insurance for COVID-19 patients.

Characteristics	Number of cases	Cost per case (USD)	The total cost of COVID-19 cases (USD)
Mild to moderate	60	2003	120,155
Sever	148	14,545	2,152,671
Critically ill	79	20,188	1,594,876
Total	287	13,476	3,867,701

## Discussion

The COVID-19 pandemic does not only cause a huge impact on the healthcare systems, but it is also a crisis that affects the economy worldwide. In assessing the pandemic's economic impact on the healthcare sector, it is essential to understand the cost of treating hospitalized COVID-19 patients. This helps with future risk preparedness, response planning, and economic evaluation of global health emergencies^3^. Pandemics disastrously impacted healthcare spending and the global economy^14^. A study in the United States estimated the potential healthcare costs associated with infected populations to be ranged between $163.4 billion to $654 billion^15^.

Our analysis showed that the average cost for treating patients infected with COVID-19 in Saudi Arabia ranged from 2003 USD for mild to moderate cases to 20,188 USD for critically ill patients managed in intensive and specialized hospital settings. Thus, the cost of COVID-19 treatment could increase up to 10-folds once a patient’s condition needs critical care. This can be explained as critically ill patients are resource-intensive. They need intensive care, expensive antiviral drugs, and oxygen support. In addition, they require more focused time from health care professionals.

Patients with comorbidities are more likely to have more severe COVID-19 disease compared to COVID-19-infected individuals without any comorbidities. Therefore, patients with comorbidities require more medications to stabilize their conditions and more intubation and monitoring procedures. Additionally, patients with comorbidities need frequent lab investigations. Given the situation of deteriorating population lifestyle, treating pandemics is going to be more costly, adding extra burden to the country’s health economics.

Only a limited number of published papers are available to measure the cost of treating COVID-19 patients, including case management, which is the focus of this article. Additionally, it is difficult to compare the literature due to differences in the study methodology, population, cost of medications, and medical equipment. In a previous study of 70 patients in China, the cost of treating COVID-19 patients was found to be 6827 USD per treated episode^16^. Moreover, this study reported that the mean cost was higher for patients with pre-existing diseases; supporting our finding. Interestingly, this study showed that the highest cost was spent on treatment medications, accounting for 45.1% of the total cost. This finding also aligned with our finding as the highest cost was observed with the treatment medications.

Association between cost and pre-existing health conditions has been reported in a study conducted in Brazil^12^. An increasing trend was observed with the number of comorbidities. Comparing patients with no comorbidities, having two or three comorbidities increased the average admission cost by 16% while having more than three comorbidities increased the cost by 19%. In comparison with our finding, the mean cost for treatment of patients with multiple comorbidities was 55% higher than patients without comorbidities.

In Saudi Arabia, the average direct medical cost per patient per day for patients with moderate-to-severe COVID-19 symptoms admitted to the general medical ward was 42,704.49 SAR (11,387.864 USD), which was lower than the average cost per patient per day for ICU patients (21,178.213 USD)^17^. The difference in the cost for ICU patients between the previous study and our study is less than 1000 USD; supporting the accuracy of our findings.

The cost of treating COVID-19 patients with different disease severities should be considered to have a clear plan for resource allocation and an emergency budget for any upcoming pandemics or epidemics. In addition, one important lesson that can be taken from the pandemic COVID-19 is that preventive measures should be taken as early as possible to avoid the extra cost of treating patients which is a huge burden on the country’s economy. Early preventive measures give time for researchers around the world to understand the pandemic and try to get a solution. As in COVID-19, a vaccine developed and disease severity started to decrease; less money was spent on treating patients.

The study covered only thirteen months of the pandemic. Therefore, we cannot capture the long-term economic effects of COVID-19. Future research is required to assess the long-term economic impact of COVID-19 on the healthcare system. Moreover, these data were only from one hospital in Saudi Arabia; affecting the generalizability of the findings. Furthermore, the number and salaries of labor, food services, and rehabilitation services were not taken into account during calculating the cost in the study which is considered a limitation of this study. However, the study aim was to measure the cost of treating COVID-19 patients as labor cost will be paid regardless of the presence of pandemic. Additionally, the private hospital where the study was conducted did not have any additional employment nor salary increment during the study period. Changes in treatment protocols and their possible impact on mortality and recovery rates were not considered in the study which is another limitation of the study.

## Conclusions

The cost of treating COVID-19 patients is considered a burden for many countries. As COVID-19 becomes more severe, treating patients becomes more costly. In addition, the presence of comorbidities increased the cost significantly compared to patients without comorbidities. More studies from different private and governmental hospitals are needed for comparison with the study findings for better preparation for the current COVID-19 as well as future pandemics.
